# Effects of a Gluten-Free Diet in a Teenager Presenting With Psychosis

**DOI:** 10.7759/cureus.41807

**Published:** 2023-07-13

**Authors:** Olfa Selmi, Banan Khalid, Saleem Al-Nuaimi

**Affiliations:** 1 Mental Health Service, Hamad Medical Corporation, Doha, QAT

**Keywords:** auto immune, gluten-free diet, psychosis, sensitivity, gluten

## Abstract

Nonceliac gluten sensitivity is a gluten-related disorder that results from immune-mediated reactions in predisposed people. It manifests usually with gastrointestinal symptoms; however, in rare cases, it might present with psychiatric symptoms that could be severe enough to impair functioning.

In this case report, we present a case of a 15-year-old girl, with no past psychiatric history, who presented to the Emergency Department (ED) with anxiety symptoms and paranoid delusion that did not improve on conventional treatment. However, a significant improvement was observed upon starting on a strict gluten-free diet (GFD). This case adds to the existing literature, suggesting a possible strong relationship between gluten ingestion and psychiatric disorders.

## Introduction

Gluten-related disorders include three main pathologies following the ingestion of gluten, resulting in an immune-mediated response: celiac disease (CD), nonceliac gluten sensitivity (NCGS), and wheat allergy (WA). Although both CD and NCGS are caused by exposure to gluten in susceptible individuals with genetic predispositions, their underlying pathogenetic mechanisms are different [1]. NCGS typically lacks gastrointestinal symptoms, villous atrophy, and antibodies present in CD [2].

NCGS has a prevalence of six times that of CD and is more associated with neurologic and psychiatric presentations such as depression, anxiety, and psychosis [1-2]. There have been numerous studies trying to explore the linkage between the ingestion of gluten and the onset of neurologic and psychiatric disorders, including schizophrenia, mood disorders, anxiety, and autism [3]. The pathogenesis of neuropsychiatric manifestations following gluten ingestion in NCGS patients is still unclear. The recent focus on an autoimmune etiology of psychotic disorders has been increasing as it was found to share several genetic features with other autoimmune diseases and has long been thought to play a role in relation to its etiology [4]. In this paper, we present the case of a 15-year-old girl who presented with paranoid delusions and anxiety symptoms that subside upon starting a gluten-free diet (GFD).

## Case presentation

A 15-year-old Sudanese girl with no prior personal psychiatric history and no current significant medical concerns presented to the Emergency Department (ED) at Hamad Medical Corporation (HMC), Doha, Qatar, in March 2021.

She presented due to concerns of agitation, behavioral changes (repeatedly tapping her head and screaming inappropriately), and reporting visual and auditory hallucinations of her father. Her father died about three months earlier. The patient’s mother reported that she noticed changes after the patient witnessed the burial of her father. Despite initially adjusting well, according to the mother, the patient started experiencing flashbacks of her time with her father as well as intrusive, intense images of her deceased father. She was preoccupied with these images and flashbacks. She started to express significant anxiety around death and dying. She started to exhibit paranoia toward family members, classmates, and teachers. She became hypervigilant and suspicious that people are looking at her and making some sort of negative judgments or are simply staring at her. She then started to express to her mother many times that people are starting to control her body and complained of thought insertion.

The patient was not on any medications and had no known drug allergies. However, she did have an allergy to a cosmetic lotion previously, atopic dermatitis, and a history of childhood asthma that did not persist.

Her mother reported that the patient’s premorbid functioning was very good as she was doing very well both socially and academically at school. There was no history of developmental problems, and the patient denied sexual activities and recreational drug use with a negative urine drug screen done at the ED. 

With regards to family history, the mother informed us that the patient’s older sister was diagnosed with an unspecified psychotic disorder and started on antipsychotic treatment with a very good response; however, we could not get further details about it. 

The patient was quite agitated and frankly psychotic in the ED. She required rapid tranquilization and stabilization. She received a combination of lorazepam 2 mg and haloperidol 2.5 mg. She was admitted to the psychiatry inpatient unit and started on olanzapine 5 mg, which was titrated to 10 mg. The patient had been on medication for over nine days with no observed improvement, leading to a switch to risperidone 2 mg at bedtime. 

Her initial blood tests showed significant hyperthyroidism with thyroid-stimulating hormone (TSH) levels of 0.01 mIU/L, free triiodothyronine (FT3) levels of 10.9 pmol/L, and FT4 levels of 35.9 pmol/L. Internal medicine was consulted, and she was found to have thyrotoxicosis due to Grave’s disease (TSH receptor antibodies were positive). She was started on a combination of carbimazole and propranolol with ongoing follow-up by the endocrinology team. The remainder of her investigations at the time were unremarkable. Despite initiating treatment with an appropriate dose of thyroid medications, there was no significant improvement in her psychiatric symptoms. Additionally, social isolation and low mood were observed, leading to the addition of escitalopram 10 mg to her treatment regimen.

The patient remained an inpatient for a total of 15 days and was discharged in early April 2021. The patient was discharged after slight improvement on the following medications: risperidone 2 mg at bedtime, escitalopram 10 mg daily, carbimazole 20 mg daily, and propranolol 10 mg TID (three times a day).

She was urgently referred to the Child and Adolescent Mental Health Services (CAMHS) at HMC for ongoing follow-up and management. She was seen two days after her discharge.

She was noted to be extremely anxious with persistent but attenuated and less prominent psychotic symptoms. Other pertinent findings were ongoing weight loss (with no appetite changes), and her labs showed ongoing asymptomatic mild elevation of prolactin levels. Clinically, it was difficult to determine if her psychiatric presentation was simply manifestations of hyperthyroidism, which she just started receiving treatment for. The plan was to decrease risperidone to 1.5 mg and continue with her other medications unchanged. Additionally, an urgent head MRI was ordered, and close follow-up appointments were scheduled to monitor her progress. Within a few days, the patient started to experience more prominent and intense anxiety and paranoid delusions so the risperidone was increased back to 2 mg again.

One week later, the patient was brought to ED again by her mother for agitated behavior at home. The patient complained about her medications at home. She exhibited guarded behavior and verbally assaulted a female security guard by yelling and insulting her. Additionally, she refused to be seen by a female physician, believing that all females were somehow against her. According to her mother, the patient had been expressing clear persecutory delusions related to her mother (such as the belief that her mother was controlling her body) and had been increasingly irritable in the past few days. No acute risk concerns were identified.

Investigations revealed an unremarkable head CT scan, and thyroid function testing showed signs of normalization. Other extensive blood tests were unremarkable. She was discharged home with the same medications and an urgent follow-up appointment.

She was reassessed at the outpatient CAMHS four days later (first on April 7, 2021). The nature of her psychotic symptoms was quite atypical and worsening. She reported olfactory hallucinations of smelling onions and garlic when no such smell was evident by other family members. Her delusions became more bizarre (e.g., believing that her mother caused a blood clot in her brain, resulting in a headache). Her paranoia worsened and started to develop more first-rank Schneiderian symptoms (passivity phenomena and running commentary hallucination). It was felt that her condition warranted more extensive investigations, including an urgent referral to neurology, further endocrinology workup, autoimmune workup, and genetic workup.

The dose of risperidone was increased to a total of 5 mg daily. The patient and her mother reported gradual improvement in her symptoms over eight weeks. However, she did not achieve remission and only exhibited a partial response. We also conducted trials of probiotics, vitamin D, B12, and omega-3 supplements, but there were no significant changes observed in the patient's condition.

She had additional workup, including blood tests, magnetic resonance imaging (MRI), and electroencephalography (EEG). Her EEG was unremarkable. The blood tests showed normalized thyroid functions. She continued to have hyperprolactinemia, which was thought to be due to risperidone. Most importantly, she was found to have positive anti-transglutaminase IgA antibodies (11.00 U/mL), while anti-transglutaminase IgG antibodies were negative (2.90 U/mL).

She underwent a brain MRI with contrast and an MRI pituitary with contrast. The summary finding of the MRI revealed a suspicious tiny hypo-enhancing low-density area within the right posterior aspect of the adenohypophysis. It was noted as a potential microadenoma and suggested the need for clinical/laboratory correlation and follow-up.

The internal medicine team and the neurology team did comment on this finding as being clinically nonsignificant.

The patient continued to have follow-up appointments with our outpatient service without much improvement. She continued to receive continuous antipsychotic medications at appropriate doses, along with carbimazole and escitalopram, for over three months. During this time, she awaited her gastroenterology appointment to further investigate the previously mentioned antibody findings.

She underwent gastroscopy in February 2022, after the aforementioned antibodies result with the following findings:

· Linear mucosal breaks <5 mm were seen at the esophagogastric junction with no significant hiatus hernia.

· No cardio incontinence was seen. Normal mucosa was seen in the fundus and body with few erosions seen in the antrum along the anterior wall. Normal mucosa seen in D1 and D2. Biopsies taken.

· The endoscopic diagnosis was gastroesophageal reflux disease and gastric erosions.

The biopsy findings are as follows:

· Duodenal mucosal fragments showed a mild and focal increase in intraepithelial lymphocytes, while the villous architecture remained intact. Additional comments are provided.

· Focal peptic duodenitis was identified.

· The biopsy was negative for parasitic infestation, granulomas, dysplasia, and malignancy.

While such appearances could be present in celiac disease, they are not diagnostic/specific. Other differential diagnoses should also be considered. Correlation with serology and endoscopic findings is recommended.

The patient went on a strict GFD and was referred to our dietician for ongoing management. The mother reported a significant and noticeable improvement *of 60%* in psychotic and anxiety symptoms within only three weeks of starting the GFD. The mother reported that the patient was no longer delusional but had overvalued ideas instead that she was said to have been a victim of black magic. She was noticeably less anxious, less agitated, and calmer. It was also reported by her mother that when the patient eats gluten in large quantities, she had a significant resurgence of delusions against the mother. This lasts for a few days and then subsided. The patient returned to her baseline along with going back to her GFD. This pattern was noticeable and replicable every time she did not stick to a GFD.

We continued a very strict GFD and optimized her medications. Her current medications include escitalopram 20 mg daily, risperidone 1 mg in the morning (AM), and 4 mg at bedtime, and levothyroxine 25 mcg daily after she was found to have hypothyroidism. Since September 2022, she has achieved complete remission of psychotic symptoms. Moreover, she has successfully returned to her pre-illness baseline in terms of social and academic functioning. She has not only resumed attending school but has also excelled academically. Additionally, she engages in appropriate social interactions, has resumed participation in team sports, and enjoys the ability to travel and visit family and friends without any observable concerns reported by others.

## Discussion

This case highlights the possible clinical impact of a GFD in reducing positive psychotic symptoms in a teenage girl who presented with complex psychiatric symptoms, including anxiety, trauma symptoms, and psychosis. These symptoms persisted despite standard therapeutic interventions involving antipsychotic and antidepressant medications. Grave's disease was well controlled, and her thyroid function had normalized. Equally significant is the observation of worsening psychosis when the patient consumed gluten while being maintained on the same dose of antipsychotic medication. 

The assumed mechanism of gluten in the pathophysiology of psychosis remains uncertain as there has been no consistency in the clinical, immunological, microbiological, and epidemiological studies investigating the relationship between psychotic disorders and gluten-related disorders [5]. However, the literature does point to a more robust link between GFD in improving positive psychotic symptoms in patients with schizophrenia and autoimmune disease [4-5]. Nonceliac gluten sensitivity is frequently comorbid with other autoimmune diseases, including type 1 diabetes, Grave’s disease, and inflammatory bowel diseases as some research suggests that inflammation may have a role in the etiology of psychotic disorders with evidence of proinflammatory activation of the innate immune system [6].

It is important to recognize how this patient could have possibly gone on to more aggressive treatments (such as clozapine and electroconvulsive treatment) and polypharmacy to achieve better symptom control. However, these approaches would have exposed the patient to a significantly higher risk of iatrogenic harm and potentially life-threatening side effects. In contrast, a GFD does not carry such concerns. Previous case reports have shown complete resolution of psychotic symptoms in patients with psychotic disorder and after going on a GFD [6-7]. Therefore, a trial of GFD may be suggested as part of the treatment plan for reducing psychotic symptoms in patients with any form of gluten allergy/sensitivity and/or comorbid autoimmune disorders. Given the low cost and safety profile of such an approach, it is worth exploring despite the limited/mixed evidence in the literature.

## Conclusions

In this report, we highlighted the role of GFD in the significant reduction of psychotic symptoms in a teenage patient diagnosed with NCGS despite standard treatment. What is also of great interest is the clear association of ingestion of gluten and the inflammatory reaction secondary to that with relapse of psychotic symptoms. This report adds to the growing literature exploring the possible role of gluten in atypical psychotic presentations as well as the role of GFD in ameliorating such presentations.
